# High TLR6 Expression Status Predicts a More Favorable Prognosis after Esophagectomy for Locally Advanced Thoracic Esophageal Squamous Cell Carcinoma

**DOI:** 10.3390/curroncol30050356

**Published:** 2023-05-04

**Authors:** Yusuke Sato, Akiyuki Wakita, Eri Maeda, Yushi Nagaki, Ryohei Sasamori, Kohei Kemuriyama, Shu Nozaki, Satoru Ito, Kaori Terata, Kazuhiro Imai, Hiroshi Nanjo, Kyoko Nomura, Yoshihiro Minamiya

**Affiliations:** 1 Esophageal Surgery, Akita University Hospital, Akita 010-8543, Japan; wakita@gipc.akita-u.ac.jp (A.W.); nagaki@med.akita-u.ac.jp (Y.N.); tanya0721@icloud.com (R.S.); kemu.kohei@gmail.com (K.K.); s.noza.doc@live.jp (S.N.); minamiya@med.akita-u.ac.jp (Y.M.); 2 Department of Thoracic Surgery, Akita University Graduate School of Medicine, Akita 010-8543, Japan; trt0605@gipc.akita-u.ac.jp (K.T.); karo@doc.med.akita-u.ac.jp (K.I.); 3 Department of Environmental Health Science and Public Health, Akita University Graduate School of Medicine, Akita 010-8543, Japan; erimaeda@med.akita-u.ac.jp (E.M.);; 4 Department of Pathology, Akita University Graduate School of Medicine, Akita 010-8543, Japan; sriona@hos.akita-u.ac.jp (S.I.); hnanjo@med.akita-u.ac.jp (H.N.)

**Keywords:** esophageal squamous cell carcinoma, ESCC, toll-like receptor, TLR6, prognosis, peptidoglycan

## Abstract

Most so-called “beneficial bacteria” in gut microbiota are Gram-positive, and TLR6 recognizes the peptidoglycan (PGN) present in their cell walls. We hypothesized that a high TLR6 expression status predicts a more favorable prognosis after esophagectomy. We used an ESCC tissue microarray (TMA) to examine TLR6 expression status in ESCC patients and to determine whether TLR6 expression status correlates with prognosis after curative esophagectomy. We also examined whether PGN influences the cell proliferation activity of ESCC lines. Clinical ESCC samples from 177 patients tested for the expression of TLR6 were categorized as 3+ (n = 17), 2+ (n = 48), 1+ (n = 68), or 0 (n = 44). High TLR6 expression (3+ and 2+) correlated with significantly more favorable 5-year overall survival (OS) and disease-specific survival (DSS) after esophagectomy than a lower TLR6 expression (1+ and 0). Univariate and multivariate analyses showed that TLR6 expression status is an independent prognostic factor that affects 5-year OS. PGN significantly inhibited the cell proliferation activity of ESCC lines. This is the first study to show that high TLR6 expression status predicts a more favorable prognosis in locally advanced thoracic ESCC patients after curative esophagectomy. PGN released from “beneficial bacteria” seems to have potential to inhibit the cell proliferation activity of ESCC.

## 1. Introduction

Esophageal cancer climbed up from the 8th to the 7th most common cancer worldwide, with 604,000 new cases and about 544,000 deaths in 2020 [1]. A geographic variation in the histological subtypes of this cancer has been elucidated. Esophageal adenocarcinoma (EAC) predominates in North America, Europe, and Oceania, while esophageal squamous cell carcinoma (ESCC) predominates in Central South America, Africa, and Asia [2,3]. The results of previous studies suggest that smoking, heavy alcohol consumption, and poor dietary habits can all contribute to the pathogenesis of ESCC [4]. More recently, it had been reported that poor oral health is an independent risk factor for upper-aerodigestive tract cancers, including ESCC [5,6]. The presence of two specific periodontal pathogens, Porphyromonas gingivalis (categorized as a red complex) and Fusobacterium nucleatum (categorized as an orange complex), appears to contribute to the development and progression of ESCC and with the prognosis in those patients [7,8].

Toll-like receptors (TLRs) were found to make up a family of pattern recognition receptors (PRRs) that recognize pathogen-associated molecular patterns (PAMPs) released from a wide range of pathogens, including viruses, fungi, yeast, parasites, and bacteria [9,10,11]. These receptors play key roles in mediating innate and antigen-specific adaptive immunity [9,10]. The three periodontal pathogens comprising the so-called “Red complex” [12,13], *Porphyromonas gingivalis*, *Tannerella forsythia*, and *Treponema denticola*, are all Gram-negative bacteria, and TLR4 recognizes the lipopolysaccharide (LPS) present in their cell walls [9,10]. We recently reported that ESCC patients exhibiting high TLR4 expression show significantly worse survival than patients expressing lower levels of TLR4 [14]. Based on these results, it appears that TLR4 signaling may contribute to the pathogenesis and progression of ESCC that chronically interacts with periodontal pathogens.

Although most periodontal pathogens are Gram-negative bacteria, “beneficial bacteria” in the gut microbiome, including Lactobacillus, butyrate-producing bacteria, and Bacillus subtilis, are all Gram-positive [15,16,17], and TLR6 recognizes the peptidoglycan (PGN) present in their cell walls [9,10]. This suggests that TLR6 may play an inhibitory role in the pathogenesis and progression of ESCC that chronically interacts with beneficial bacteria. It has been reported that TLR6 expression is gradually upregulated in EAC [18], but the relationship between TLR6 expression and the pathogenesis, progression, and prognosis of ESCC remains to be elucidated. Therefore, in this study, we hypothesized that a high TLR6 expression status predicts a more favorable prognosis after curative esophagectomy and examined that relationship by assessing TLR6 expression status in clinical ESCC samples. In addition, we also tested whether the combined expression of TLR6 and TLR4 correlates even more closely with 5-year overall survival (OS) and disease-specific survival (DSS) in these patients. We also examined whether PGN influences the cell proliferation activity of ESCC lines to prove whether PGN released from Gram-positive bacteria inhibits ESCC progression.

## 2. Materials and Methods

### 2.1. Patients

This study was approved (#2324) by the Ethics Committee of Akita University School of Medicine on 20 November 2019. All the experiments were performed in accordance with the Helsinki Declaration. All study participants provided informed written consent. Between January 2000 and December 2011, 507 patients received esophagectomy to treat esophageal cancer at Akita University Hospital. Among them, 177 patients with pT2-pT4 thoracic ESCC who had received curative esophagectomy with no preoperative treatment were enrolled in this study [19]. The clinical cancer stages and treatment strategies were discussed and decided by a cancer board composed of radiologists, oncologists, gastroenterologists, and surgeons. Pathological stages were determined according to the UICC International Union Against Cancer Tumor–Node–Metastasis (TNM) Classification of Malignant Tumors (8th edition).

### 2.2. Esophagectomy

Our standard operative procedure was right thoracoscopic or robot-assisted thoracoscopic esophagectomy with extended three-field lymph node dissection. The three fields included: (1) mediastinal fields, involving the periesophageal region and areas around the trachea and bilateral main bronchus; (2) abdominal fields, involving the perigastric region and areas around the celiac axis; and (3) cervical fields involving the bilateral periesophageal region and the supraclavicular region. Reconstruction often involved the insertion of a gastric conduit via the posterior mediastinal route or the retrosternal route [19,20].

### 2.3. ESCC Tissue Microarray

An ESCC tissue microarray (TMA) was constructed at the Pathology Institute, Toyama, Japan, as previously described [14,19,20,21]. According to many validations of TMA to overcome cancer tissue heterogeneity, an assessment of double cores measuring 0.6 mm in diameter sufficiently reflected the whole section [22]. We therefore employed triplicate cores measuring 0.6 mm in diameter to further enhance the reliability. Triplicate cores were randomly collected from separate carcinoma areas and transferred to the TMA. The TMA block contained 531 cores (3 cores each from 177 paraffin main tumor blocks from enrolled ESCC patients) in total.

### 2.4. Immunohistochemistry (IHC)

Sections (4 μm) from the TMA were immunohistochemically examined using standard procedures [14,19,20,21]. Briefly, sections were incubated in a citrate buffer (pH 6.0) for 10 min at 121 °C in an autoclave for antigen retrieval purposes. Endogenous peroxidase activity was inactivated by incubation for 5 min in 3.0% H_2_O_2_, and internal biotin was blocked using a biotin-blocking system (Dako, Denmark). Then, the sections were incubated with an anti-TLR6/CD286 monoclonal antibody (10 μg/mL; IMG-304A, IMGENEX, San Diego, CA, USA) overnight at 4 °C, after which the antigen was detected using a catalyzed signal amplification system (Dako) with a DAB peroxidase substrate. Finally, the sections were counterstained with Mayer’s hematoxylin, dehydrated, and mounted.

IHC staining was imaged and viewed using a NanoZoomer Digital Pathology C9600 slide scanner and Virtual Slide Viewer software (NDP.view2 version 2.9.29) (Hamamatsu Photonics, Hamamatsu, Japan). Three physicians blinded to clinical and prognostic data assigned a staining score. We employed the HER2 IHC scoring system (ASCO and CAP guidelines) [23]. A sample was assigned an IHC score of 3+ if there was intense TLR6 staining in the cytoplasm or nuclei in more than 30% cells, 2+ if there was moderate staining in the cytoplasm or nuclei in >10% of cells, 1+ if there was weak staining, or 0 if there was no staining. High expression was defined as an IHC score of 3+ or 2+; low expression was defined as a score of 1+ or 0. If the scoring was not unanimous, the score assigned by 2 of 3 physicians was adopted. A database of IHC scores of TLR4 staining in the same cohort from an earlier study was used [14]. The study was performed in accordance with the REMARK criteria [24].

### 2.5. Cell Lines

Two SCC lines, KYSE190 and OE21, were studied. KYSE190 was obtained from the Health Science Research Resources Bank (Osaka, Japan). OE21 was obtained from the European Collection of Cell Cultures. All cell lines were cultured in RPMI1640 (Sigma-Aldrich, St Louis, MO, USA) and supplemented with 10% (*v*/*v*) heat-inactivated fetal bovine serum (FBS) (GIBCO, Grand Island, NY, USA) and antibiotics (penicillin G–streptomycin–amphotericin B, GIBCO). These lines were maintained in a humidified incubator under 5% CO_2_/95% air at 37 °C.

### 2.6. Cell Proliferation Assay

The effects of PGN on the cell proliferation activity of ESCC lines were examined. Cells were plated to a density of 1 × 10^3^ cells/well in 96-well plates and incubated for 24 h in 100 μL of RPMI1640. After being washed with PBS, the cells were incubated for an additional 72 h in 100 μL of RPMI1640 alone (control) or with 10 ng/mL of PGN (69554 Sigma-Aldrich, Saint Louis, MO, USA). Thereafter, the cell numbers were determined using a CellTiter-Glo Luminescent Cell Viability Assay Kit (Promega, Madison, WI, USA), in accordance with the manufacturer’s protocol. The average of the control wells was defined as 100%. Each sample was analyzed in 8 wells, after which the data were expressed as means ± SD and compared to the control.

### 2.7. Statistical Analysis

Continuous variables are presented as the median (range: minimum–maximum). To assess the differences between the TLR6-high and TLR6-low groups, the Mann–Whitney–Wilcoxon test (for continuous variables) or χ^2^ and Fisher’s exact tests (for categorical variables) were performed. The clinical end-points of this study were OS and DSS. The length of survival (censored at 60 months for longer survivors) was calculated from the date of esophagectomy to the patient’s death or the date of the last clinical follow-up. The Kaplan–Meier method was used to construct OS and DSS curves, which were compared using the log-rank test. Cox’s proportional hazards model was used to assess whether TLR6 expression is predictive of a favorable survival among clinical ESCC samples. The variables adjusted for in multivariate analysis were age, sex, pT, pN, pStage, and tumor differentiation. All statistical analyses were performed using JMP Pro 14.2.0. Values of *p* < 0.05 were considered to be statistically significant.

## 3. Results

### 3.1. Immunohistochemical Analysis of TLR6 Expression

**Figure 1:**
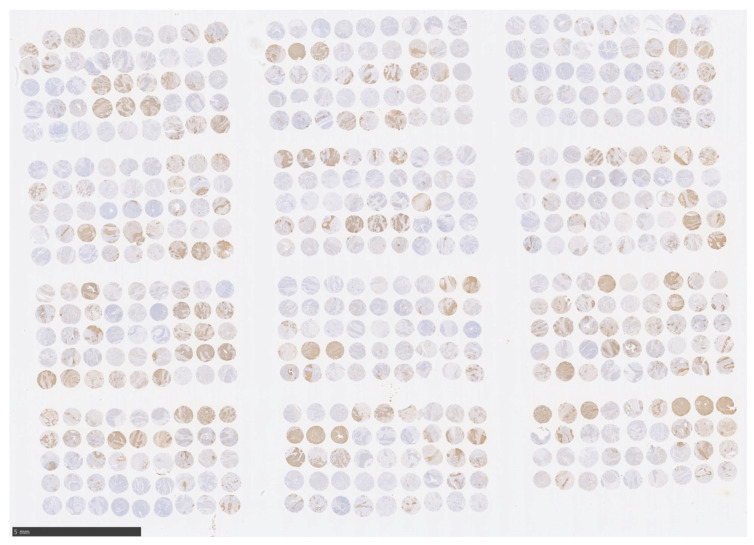
Images showing entire ESCC tissue microarray immunohistochemically stained for TLR6. The TMA block contains 531 cores (3 cores each (0.6 mm in diameter) from 177 paraffin blocks); 27 cores (3 cores each from 9 patients) are sorted horizontally from left to right (scale bar: 5 mm).

**Figure 2:**
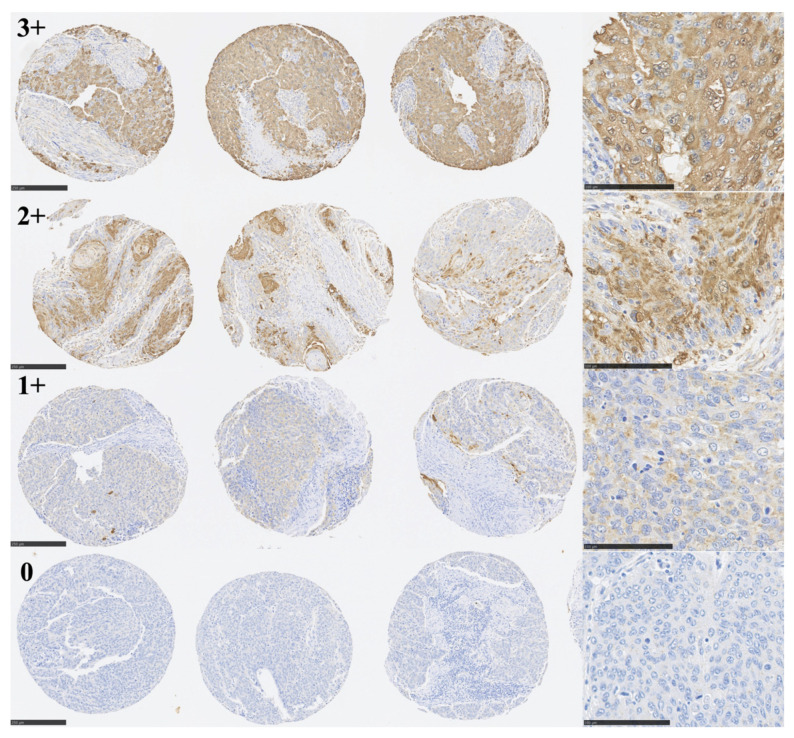
Immunohistochemical staining for TLR6 in selected ESCC cores. Photomicrographs of representative triplicate specimens, scored as 3+, 2+, 1+, or 0, are shown. The triplicate cores are shown at 100× *g* magnification (scale bar: 250 μm), along with high-magnification (400×) images on the right (scale bar: 100 μm).

An image of the entire ESCC TMA that was immunohistochemically stained for TLR6 is shown in Figure 1. Three representative cores, each assigned IHC scores of 3+, 2+, 1+, or 0, are shown in Figure 2. Specimens scored as 3+, 2+, 1+, or 0 were obtained from 17 patients (9.6%), 48 patients (27.1%), 68 patients (38.4%), and 44 patients (24.9%), respectively. We considered scores of 3+ and 2+ to be TLR6-high (n = 65), while 1+ and 0 were considered to be TLR6-low (n = 112). The clinicopathological characteristics of the two groups are summarized in Table 1. The TLR6-high group contained significantly more pT4a, pN0, well-differentiated tumors, and living patients. There were no significant differences between the groups with respect to other factors shown in the table.

### 3.2. TLR6 Expression Status and 5-Year OS and SDD in 177 ESCC Patients

**Figure 3:**
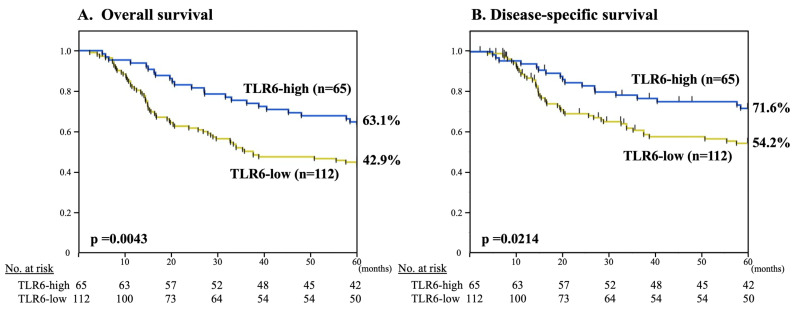
Kaplan–Meier curves illustrating the association between TLR6 expression status (high or low) and 5-year OS (**A**) and DSS (**B**) in ESCC patients after curative esophagectomy.

All 89 censored cases were followed up for 60 months. Kaplan–Meier curves illustrating the association between TLR6 expression status and survival are shown in Figure 3. High TLR6 expression was associated with significantly better 5-year OS and DSS (*p* = 0.0043 and *p* = 0.0214, respectively).

### 3.3. TLR6 Expression Status Is a Prognostic Factor Affecting 5-Year OS

Significant prognostic factors affecting 5-year OS with univariate analyses were pN status (N0 vs. N1-3), pStage (Stage IIA-IIIA vs. over IIIB), tumor differentiation (poorly vs. not poorly differentiated), and TLR6 expression status (low vs. high) (Table 2). Moreover, multivariate analysis proved that TLR6 expression status is an independent prognostic factor in every combination with age, sex, pT, pN, pStage, and tumor differentiation (Table 2).

### 3.4. Combined TLR6 and TLR4 Expression Statuses and 5-Year OS and DSS

**Figure 4:**
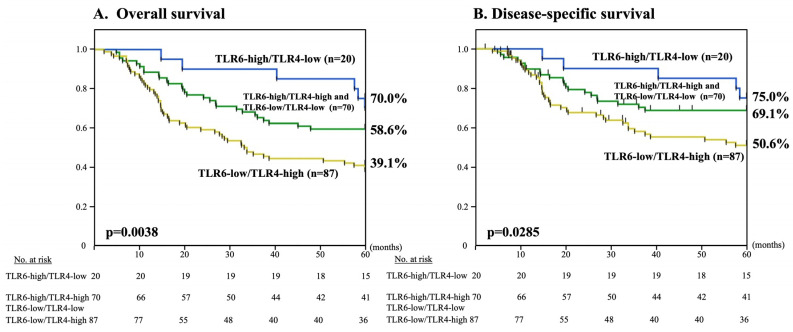
Kaplan–Meier curves illustrating the association between the combined TLR6 and TLR4 expression statuses (TLR6-high/TLR4-low, TLR6-high/TLR4-high and TLR6-low/TLR4-low, and TLR6-low/TLR4-high,) and 5-OS (**A**) and DSS (**B**) in ESCC patients after curative esophagectomy.

Our previous data of the two groups, TLR4-high (n = 132) and TLR4-low (n = 45), are summarized in Supplementary Table S1 [14]. In total, 25 patients exhibited combined TLR6-low/TLR4-low expression (14.1%), 87 patients exhibited TLR6-low/TLR4-high expression (49.2%), 20 patients exhibited TLR6-high/TLR4-low expression (11.3%), and 45 patients exhibited TLR6-high/TLR4-high expression (25.4%). There was no statistical correlation between TLR6 and TLR4 expression statuses (*p* = 0.21). Patients exhibiting combined TLR6-low/TLR4-high expression had much worse 5-year OS and DSS than other patients (*p* = 0.0038 and *p* = 0.0285, respectively) (Figure 4). Consistent with the results of 5-year OS and DSS, the patient survival rate was twice as high among TLR6 high/TLR4-low patients (60.0%) than among TLR6 low/TLR4-high patients (33.3%) (Table 3).

### 3.5. The Effect of PGN on the Cell Proliferation Activity of ESCC Lines

**Figure 5:**
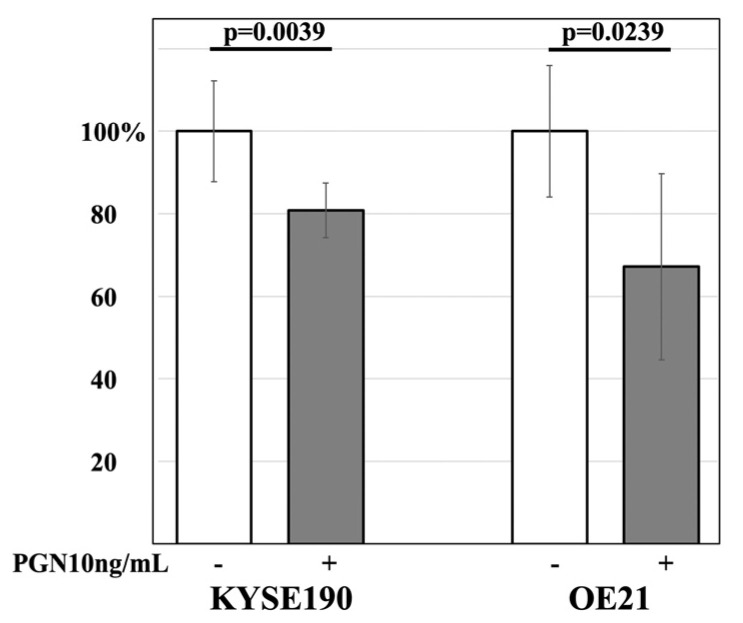
KYSE190 and OE21 showed significantly decreased cell proliferation activity after 10 ng/mL of PGN treatment compared to the control (*p* = 0.0039 and *p* = 0.0239, respectively).

The effect of PGN, an agonist of TLR6, on the cell proliferation activity of KYSE190 and OE21 was examined using the cell proliferation assay. Both lines showed significantly decreased cell proliferation activity after being treated with 10 ng/mL of PGN compared to the control (*p* = 0.0039 and *p* = 0.0239, respectively) (Figure 5).

## 4. Discussion

In this study, we elucidated that, among patients treated with curative esophagectomy for ESCC, those exhibiting high TLR6 expression had significantly better 5-year OS and DSS than those with low TLR6 expression. Moreover, multivariate analysis clearly proved that TLR6 expression status was an independent prognostic factor affecting 5-year OS. In addition, patients exhibiting TLR6-low/TLR4-high expression exhibited much poorer 5-year OS and DSS than other patients. We also showed that PGN significantly inhibited the cell proliferation activity of ESCC lines.

There have been few reports addressing the relationship between TLR6 signaling and cancer progression or prognosis. TLR6 expression was previously reported to be significantly lower in colon cancer than normal colon tissue [25], which is consistent with the idea that TLR6 signaling exerts an inhibitory effect on carcinogenesis. It was also reported that Lactobacillus-induced TLR6 signaling reduced tumor burdens and suppressed inflammation in inflammation-induced colorectal cancer [26]. More recently, it was revealed that Lactobacillus animalis, a type of Lactobacillus, was negatively associated with oral carcinogenesis in mice [27]. These results are also consistent with the idea that TLR6 signaling exerts an inhibitory effect on carcinogenesis. Based on these data, a relationship between PGN released from Gram-positive “beneficial bacteria”, TLR6 signaling exertion, and inhibitory effects on carcinogenesis is hypothesized.

In this study, the TLR6-high group contained significantly more pT4a patients than the TLR6-low group. However, the TLR6-high group also contained significantly more pN0 patients and a significantly more favorable prognosis after esophagectomy than the TLR6-low group. This suggests that TLR6 signaling may have an inhibitory effect on lymph node metastasis in ESCC, which correlates with prognosis. Semlali et al. examined the relationship between TLR6 SNPs and breast cancer and indicated that these SNPs had a neutral effect within the TLR6 structure and a protective effect against breast cancer risk [28]. These SNPs cause TLR6 to function abnormally, leading to the induction of breast cancer. This result provides further evidence on the inhibitory effect of TLR6 signaling on carcinogenesis.

As mentioned, we previously reported that high TLR4 expression has a negative effect on 5-year OS and DSS [14]. Most periodontal pathogens are Gram-negative bacteria, and TLR4 recognizes the LPS present in their cell walls. In this study, we showed that both TLR6 and TLR4 expression statuses have the potential to reveal patients with a poor prognosis after esophagectomy—i.e., those exhibiting TLR6-low/TLR4-high expression. This highlights the importance of oral bacterial flora. A large body of evidence has demonstrated that alterations in oral and gut bacterial flora are key factors that contribute to the pathogenesis of a variety of local and systemic disorders, including periodontitis, obesity, diabetes, cerebral infarction, myocardial infarction, Alzheimer’s disease, premature birth, osteoporosis, and various cancers [29,30]. We also previously reported that about 80% of ESCC patients in a Japanese population were diagnosed with periodontitis, and about half of that group required dental extraction before the cancer treatment [31]. Thus, pathogenic bacteria dominate the oral flora in most ESCC patients. It is unequivocal that esophageal epithelial cells are directly and heavily affected by oral bacterial flora because it flows into the esophagus with every swallow. Therefore, correcting the oral bacterial flora by replacing pathogenic bacteria with “beneficial bacteria” could have a potentially preventative or suppressive effect on the development and progression of ESCC. It is also noteworthy that where risk factors for oral cancer are present, the use of mouthwashes containing alcohol or other anti-bactericidal agents may further increase the risk of developing oral cancer [32]. This may reflect a situation in which mouthwashes containing alcohol or other anti-bactericidal agents remove both pathogenic and beneficial oral bacteria, upsetting the balance of TLR6 and TLR4 signaling and potentially increasing the risk of cancer development.

There are some limitations in this study. The most important limitation is the lack of data on the status of the patients’ oral cavity environment and an assessment of oral bacterial flora before esophagectomy. Because we started a preoperative assessment of the oral cavity environment monitored by dentists before esophagectomy in 2009 [31], those data were not available for most patients in the cohort used for this study. Whether the oral cavity environment or its bacterial flora are associated with TLR6 expression status and prognosis remains to be determined. Moreover, demonstrating a direct relationship between TLR6 signaling and ESCC progression or lymph node metastasis requires extensive in vitro and in vivo studies in the future. In this study, we could only show the inhibitory effect of PGN on the cell proliferation activity of ESCC lines in an in vitro study. The mechanisms of this inhibitory effect and the relation to prognosis through TLR6 signaling mediators and cytokines remain to be determined in future studies. Another limitation causes the cognitive ability of TLRs. Recent evidence indicates that, in addition to PAMPs, TLRs recognize damage-associated molecular patterns (DAMPs), which are endogenous molecular patterns released from injured or dying host cells (9–11). Therefore, there is a possibility that TLR6 not only recognizes PGN, but also DAMPs from host cells. The validation of this possibility also requires further studies.

## 5. Conclusions

This is the first study to show that high tumoral TLR6 expression is predictive of a more favorable prognosis in patients receiving curative esophagectomy for locally advanced thoracic ESCC. PGN released from “beneficial bacteria” seems to have the potential to inhibit the cell proliferation activity of ESCC. Based on these results, we could speculate that correcting the oral bacterial flora might be a useful strategy for improving the outcomes of patients after curative esophagectomy for ESCC.

## Figures and Tables

**Table 1:** The clinicopathological characteristics of 177 ESCC patients.

Characteristics	TLR6-High n = 65 (36.7%)	TLR6-Low n = 112 (63.3%)	*p* Value
**Sex** Female Male			0.082
	5 (7.7%)	19 (17.0%)	
	60 (92.3%)	93 (83.0%)	
**Age at surgery**	65 (50–76)	67 (38–82)	0.052

**Smoking history** (pack/day × year)	40 (0–120)	40 (0–250)	0.327
**Habitual smoking** Current			0.804
	36 (55.4%)	64 (57.1%)	
Past Never	16 (24.6%) 13 (20.0%)	23 (20.6%) 25 (22.3%)	
**Habitual alcohol consumption** Current Past Never	48 (73.9%) 9 (13.9%) 8 (12.2%)	84 (75.0%) 14 (12.5%) 14 (12.5%)	0.968
**Tumor location** Upper Middle Lower	2 (3.1%) 47 (72.3%) 16 (24.6%)	4 (3.6%) 69 (61.6%) 39 (34.8%)	0.345
**Depth of invasion (pT)** pT2 pT3 pT4a	10 (15.4%) 48 (73.8%) 7 (10.8%)	21 (18.7%) 89 (79.5%) 2 (1.8%)	0.031 *
**Lymph node metastasis (pN)** pN0 pN1 pN2 pN3 M1 Lymph (supraclavicular)	26 (40.0%) 18 (27.7%) 8 (12.3%) 8 (12.3%) 5 (7.7%)	23 (20.6%) 37 (33.0%) 27 (24.1%) 12 (10.7%) 13 (11.6%)	0.047 *
**Pathological stage** pStage IIA pStage IIB pStage IIIA pStage IIIB pStage IVA pStage IVB (M1 Lymph)	9 (13.9%) 15 (23.1%) 1 (1.5%) 25 (38.4%) 10 (15.4%) 5 (7.7%)	11 (9.8%) 12 (10.7%) 6 (5.4%) 57 (50.9%) 13 (11.6%) 13 (11.6%)	0.123
**Tumor differentiation** Well Moderate Poor	21 (32.3%) 32 (49.2%) 12 (18.5%)	8 (7.1%) 59 (52.7%) 45 (40.2%)	<0.001 *
**Adjuvant chemotherapy** Positive Negative	42 (64.6%) 23 (35.4%)	63 (56.3%) 49 (43.7%)	0.275
**Recurrence of ESCC** Positive Negative	28 (43.1%) 37 (56.9%)	52 (46.4%) 60 (53.6%)	0.666
**Prognosis** Alive Deceased from ESCC Deceased from other cancer Deceased from other diseases	38 (58.4%) 20 (30.8%) 0 7 (10.8%)	38 (33.9%) 47 (42.0%) 5 (4.5%) 22 (19.6%)	0.007 *

* Considered significant.

**Table 2:** (A) Hazard ratios for 5-year OS: results of univariable Cox PH model analyses; (B) hazard ratios for 5-year OS associated with TLR6 expression status: results of multivariable Cox PH model analyses.

(A)			
**Variable**	***p* Value**	**Hazard Ratio**	**95% CI**
**TLR6 expression**: low (n = 112) vs. high (n = 65)	0.0052 *	1.955	1.222–3.128
**Age**: 65 and older (n = 104) vs. younger (n = 73)	0.1174	1.415	0.916–2.185
**Sex**: male (n = 153) vs. Female (n = 24)	0.1047	1.825	0.882–3.776
**Smoking history**: 40 over (n = 93) vs. under 40 (n = 84)	0.067	1.486	0.973–2.269
**Habitual smoking**: current (n = 100) vs. others (n = 77)	0.8729	1.035	0.678–1.581
**Habitual alcohol consumption**: current (n = 132) vs. others (n = 45)	0.8906	1.035	0.634–1.690
**pT**: pT3-4 (n = 146) vs. T2 (n-31)	0.4325	1.257	0.710–2.227
**pN**: pN1-3 (n = 128) vs. pN0 (n = 49)	<0.0001 *	5.77	2.785–11.95
**pStage**: IIIB over (n = 123) vs. under IIIA (n = 54)	<0.0001 *	5.162	2.667–9.991
**Tumor differentiation**: poor (n = 57) vs. others (n = 120)	0.0032 *	1.9	1.240–2.911
**Adjuvant chemotherapy**: negative (n = 72) vs. positive (n = 105)	0.4269	1.187	0.778–1.809
**(B)**			
**Variable**	***p* Value**	**Hazard Ratio**	**95% CI**
**TLR6 expression** (crude)	0.0052 *	1.955	1.222–3.128
**Adjusted for age and sex**	0.0026 *	2.07	1.289–3.322
**Adjusted for age, sex, pT, pN, pStage, and tumor differentiation**	0.0277 *	1.745	1.062–2.864

* Considered significant. CI: confidence interval.

**Table 3:** Patient outcomes taking into consideration TLR6 and TLR4 expression statuses.

	n (%)	Alive	Deceased from ESCC	Deceased from Other Cancers	Deceased from Other Diseases
**TLR6-high/TLR4-low**	20 (11.3%)	12 (60.0%)	6 (30.0%)	0	2 (10.0%)
**TLR6-high/TLR4-high** **and TLR6-low/TLR4-low**	70 (39.5%)	35 (50.0%)	23 (32.9%)	0	12 (17.1%)
**TLR6-low/TLR4-high**	87 (49.2%)	29 (33.3%)	38 (43.7%)	5 (5.8%)	15 (17.2%)

## Data Availability

The data presented in this study are available on request from the corresponding author.

## Supplementary Materials

The following supporting information can be downloaded at: https://www.mdpi.com/article/10.3390/curroncol30050356/s1. Table S1: The clinicopathological characteristics of 177 ESCC patients.
