# Supplementary material for: High TLR6 Expression Status Predicts a More Favorable Prognosis after Esophagectomy for Locally Advanced Thoracic Esophageal Squamous Cell Carcinoma
Source: Curr Oncol. 2023 May 4;30(5):4724–35. doi: 10.3390/curroncol30050356 (PMC10217459; doi:10.3390/curroncol30050356)
Supplement: Supplementary file 1 [file curroncol-30-00356-s001.zip › curroncol-2314706-supplementary.pdf]

Supplement Table S1. The clinicopathological characteristics of 177 ESCC patients

Characteristics	TLR4-high (n=132) (74.6%)	TLR4-low (n=45) (25.4%)	P value
**Sex**			1.000
**Female**	18 (13.6%)	6 (13.3%)	
**Male**	114 (86.4%)	39 (86.7%)	
**Age at surgery**	64.7±8.3 (38-78)	65.9±8.1 (38-82)	0.529
**Smoking history** (pack/day x year)	38.4±34.4 (0-250)	32.8±24.6 (0-120)	0.395
**Habitual Smoking**			0.830
**Current**	75 (56.8%)	25 (55.6%)	
**Past**	30 (22.7%)	9 (20.0%)	
**Never**	27 (20.5%)	11 (24.4%)	
**Habitual alcohol consumption**			0.097
**Current**	99 (75.0%)	33 (73.3%)	
**Past**	20 (15.2%)	3 (6.7%)	
**Never**	13 (9.8%)	9 (20.0%)	
**Tumor location**			0.881
**Upper**	5 (3.8%)	1 (2.2%)	
**Middle**	86 (65.1%)	30 (66.7%)	
**Lower**	41 (31.1%)	14 (31.1%)	
**Depth of invasion (pT)**			0.189
**pT2**	22 (16.7%)	9 (20.0%)	
**pT3**	101 (76.5%)	36 (80.0%)	
**pT4a**	9 (6.8%)	0	
**Lymph node metastasis (pN)**			0.552
**pN0**	34 (25.8%)	15 (33.3%)	
**pN1**	42 (31.8%)	13 (28.9%)	
**pN2**	28 (21.2%)	7 (15.6%)	
**pN3**	13 (9.9%)	7 (15.6%)	
**M1 Lymph (supraclavicular)**	15 (11.3%)	3 (6.6%)	
**Pathological stage**			0.746
**IIA**	14 (10.6%)	6 (13.3%)	
**IIB**	18 (13.7%)	9 (20.2%)	
**IIIA**	5 (3.8%)	2 (4.4%)	
**IIIB**	64 (48.5%)	18 (40.0%)	
**IVA**	16 (12.1%)	7 (15.5%)	
**pStage IVB (M1 Lymph)**	15 (11.3%)	3 (6.6%)	
**Tumor differentiation**			0.584

**Not poorly**	91 (68.9%)	29 (64.4%)	
**Poorly**	41 (31.1%)	16 (35.6%)	
**Adjuvant chemotherapy**			0.600
**Positive**	80 (60.6%)	25 (55.6%)	
**Negative**	52 (39.4%)	20 (44.4%)	
**Recurrence of ESCC**			0.605
**Positive**	58 (43.9%)	22 (48.9%)	
**Negative**	74 (56.1%)	23 (51.1%)	
**Prognosis**			0.270
**Alive**	55 (41.7%)	21(46.7%)	
**Deceased with ESCC**	52 (39.4%)	15 (33.3%)	
**Deceased with other Cancer**	5 (3.8%)	0	
**Deceased with other diseases**	20 (15.1%)	9 (20.0%)	
